# Sensitivity of *Oncomelania hupensis* to Niclosamide: A Nation-Wide Survey in China

**DOI:** 10.3390/ijerph110303086

**Published:** 2014-03-12

**Authors:** Jianrong Dai, Youzi Li, Wei Wang, Yuntian Xing, Guoli Qu, Yousheng Liang

**Affiliations:** 1 Jiangsu Institute of Parasitic Diseases, 117 Yangxiang, Meiyuan, Wuxi 214064, Jiangsu, China; E-Mails: djr0008@163.com (J.D.); muziyouzi@sina.cn (Y.L.); xingyuntianok@163.com (Y.X.); quguoli83@163.com (G.Q.); 2 Jiangsu Provincial Key Laboratory of Molecular Biology of Parasites, 117 Yangxiang, Meiyuan, Wuxi 214064, Jiangsu, China; 3 Key Laboratory on Technology for Parasitic Disease Prevention and Control, Ministry of Health, 117 Yangxiang, Meiyuan, Wuxi 214064, Jiangsu, China

**Keywords:** *Oncomelania hupensis*, *Schistosoma japonicum*, niclosamide, molluscicidal activity, China

## Abstract

Schistosomiasis japonica, transmitted by the intermediate host snail *Oncomelania hupensis* of the causative agent *Schistosoma japonicum*, remains a major public-health concern in China, and control of this snail is one of the major approaches used in attempts to interrupt the transmission of this neglected tropical disease. Niclosamide is currently the only commercial molluscicide available for the control of *O. hupensis* snails in China. The purpose of this study was to evaluate the current sensitivity of *O. hupensis* to niclosamide in China. *O. hupensis* snails derived from 17 sampling sites from eight schistosomiasis-endemic provinces of China were used for the molluscicidal tests. Active adult snails (10 for each drug concentration), were immersed in solutions of 1, 0.5, 0.25, 0.125, 0.063, 0.032, 0.016 and 0.008 mg/L of 50% wettable powder of niclosamide ethanolamine salt (WPN) for 24 and 48 h at 25°C, and then the snail mortality was estimated and LC_50_ values were calculated. All field-derived *O. hupensis* snails were dead following immersion in 0.5 and 1 mg/L WPN for 24 h, whereas no death was observed after immersion in 0.008 mg/L WPN for 24 h. Immersion in 0.5, 0.25, 0.125, 0.063, 0.032 and 0.016 mg/L WPN for 24 h resulted in 80%–100%, 63.33%–100%, 0%–85%, 0%–50%, 0%–15%, and 0%–5% snail mortalities, respectively. The 24 h WPN LC_50_ values for the *O. hupensis* snails derived from the 17 sampling sites in China ranged from 0.0743 to 0.2285 mg/L, and no significant difference was detected by the Kolmogorov-Smirnov test (*p* = 0.2). The results indicate that there is no regional variation in the current susceptibility to niclosamide in *O. hupensis* populations in China. It is suggested that the current sensitivity of niclosamide against *O. hupensis* remains high and has not changed after more than two decades of repeated, extensive application for snail control in the main endemic areas of China.

## 1. Introduction

Schistosomiasis, a snail-transmitted parasitic disease, is a major neglected tropical disease affecting more than 207 million people in the tropical and subtropical regions around the world [1]. Although great success has been achieved in its control in China, schistosomiasis japonica is still one of the four communicable diseases that have been given high priority by the central government [2,3,4]. It is estimated that over 0.7 million people are infected with the parasite in China, and the snail host *Oncomelania hupensis* is detected in habitats with an area of 3.73 billion m^2^ [5,6,7]. It has been proved that the transmission of *S. japonicum* is governed by the geographical distribution of its snail host; therefore, control of the *O. hupensis* snails, as a major part of the National Schistosomiasis Control Program, is currently one of the major approaches used for schistosomiasis control and elimination in China [8,9,10].

In China, many approaches have been used for the control of *O. hupensis*; however, snail control with chemicals remains the most widely used method to kill the snail intermediate host till now [11,12,13]. Niclosamide is recommended by the WHO as the only molluscicide for snail control in the endemic foci [14]; however, the agent is difficult to dissolve in both water and organic solvents. Therefore, many novel niclosamide formulations have been developed attempting to improve its water solubility. Of these a 50% wettable powder of niclosamide ethanolamine salt (WPN) has been recommended as the only market-available molluscicide since the initiation of the World Bank Loan Project for Schistosomiasis Control in China, due to its high efficacy and easy use [15]. Currently, WPN is recommended by the Ministry of Health, P. R. China at a dose of 1 mg/L for snail control in the field [15]. Following extensive, long-term, repeated use for more than two decades, the possible emergence of resistance to niclosamide in the intermediate host snails has received much attention [16]. Therefore, a systematic survey of the molluscicidal activity of niclosamide against *O. hupensis* would be of great significance for understanding the current sensitivity of niclosamide in China, and the prevention and rapid management of niclosamide resistance in snail populations. Here, a nationwide determination was carried out in regions where *O. hupensis* snails are present in China to assess the current efficacy of *O. hupensis* to niclosamide.

## 2. Materials and Methods

### 2.1. Snails

**Figure 1:**
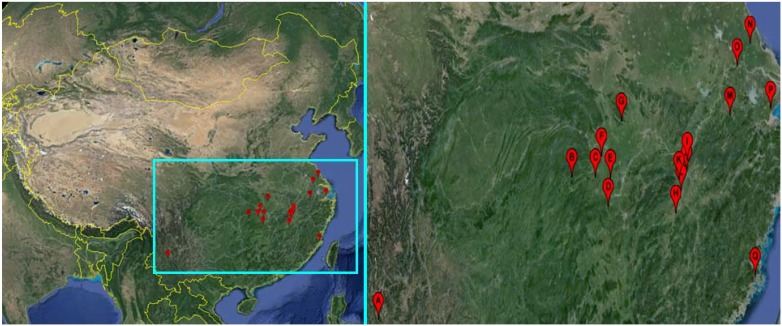
The sampling sites of *Oncomelania hupensis* snails in China.

Adult *O. hupensis* snails were collected by individual picking with forceps [17], from 17 sampling sites from eight schistosomiasis-endemic provinces of China (Table 1, Figure 1). After feeding in the laboratory for 24 h, active adult snails with 7–8 spirals were randomly divided into groups for the molluscicidal test.

**Table 1:** Location and environmental types of the snail sampling sites in China

Snail Sampling Site	Environmental Type	Code	East Longitude	North Latitude
Weishan County, Yunnan Province	Hill	A	100.33°	25.23°
Shimen County, Hunan Province	Hill	B	110.47°	29.51°
Linli County, Hunan Province	Hill	C	111.64°	29.44°
Yuanjiang County, Hunan Province	Lake and marshland	D	112.20°	28.52°
Nanxian County, Hunan Province	Lake and marshland	E	112.39°	29.37°
Gong’an County, Hubei Province	Lake and marshland	F	112.00°	30.05°
Jingshan County, Hubei Province	Hill	G	113.11°	31.03°
Wucheng County, Jiangxi Province	Lake and marshland	H	115.54°	28.05°
Pengze County, Jiangxi Province	Lake and marshland	I	116.32°	29.58°
Duchang County, Jiangxi Province	Lake and marshland	J	116.24°	29.25°
Yongxiu County, Jiangxi Province	Lake and marshland	K	115.82°	29.04°
Nanchang County, Jiangxi Province	Lake and marshland	L	115.89°	28.68°
Xuancheng County, Anhui Province	Hill	M	118.77°	30.74°
Dongtai County, Jiangsu Province	Plain with waterway networks	N	120.31°	32.84°
Zhenjiang County, Jiangsu Province	Plain with waterway networks	O	119.44°	32.20°
Pinghu County, Zhejiang Province	Plain with waterway networks	P	121.02°	30.70°
Yinxi County, Fujian Province	Hill	Q	119.35°	25.72°

### 2.2. Niclosamide Formulation

WPN was provided by the Nanjing Essence Fine Chemical Co., Ltd. (Nanjing, China; lot: 1012031). WPN was weighed and solutions of 1, 0.5, 0.25, 0.125, 0.063, 0.032, 0.016 and 0.008 mg/L of niclosamide were prepared in dechlorinated tap water for the subsequent experiments.

### 2.3. Molluscicidal Test

One hundred milliliter flasks were filled with the solutions, then 10 active adult snails were added and the flasks covered with gauze to prevent their escape. Snails in flasks of dechlorinated water served as controls. After being immersed for 24 and 48 h at 25°C, the snails were washed with dechlorinated water and fed for a further 48 h. Those suspected of being dead were tested by the knocking method [18,19], and the snail mortality was estimated. All tests were performed in triplicate, and LC_50_ values were calculated. 

### 2.4. Statistical Analysis

All data were double entered into Microsoft Excel 2003 (Microsoft Corporation; Redmond, WA, USA) and all statistical analyses were performed using the statistical software SPSS version 16.0 (SPSS Inc.; Chicago, IL, USA). Differences of LC_50_ values were tested for statistical significance with Kolmogorov-Smirnov test. A *p* value < 0.05 was considered statistically significant.

## 3. Results

All field-derived *O. hupensis* snails were dead following immersion in 1 mg/L WPN for 24 h, whereas no death was observed after immersion in 0.008 mg/L WPN for 24 h. Immersion with WPN at concentrations of 0.5, 0.25, 0.125, 0.063, 0.032 and 0.016 mg/L for 24 h resulted in 80%–100%, 63.33%–100%, 0%–85%, 0%–50%, 0%–15%, and 0%–5% mortalities of snails, respectively (Table 2). The 24 h WPN LC_50_ values for the *O. hupensis* snails derived from the 17 sampling sites in China ranged from 0.0743 to 0.2285 mg/L, and no significant difference was detected by the Kolmogorov-Smirnov test (*p* = 0.2). 

**Table 2:** Comparison of mortality rates and LC_50 _values of *Oncomelania hupensis* snails sampled from different regions of China following immersion in various concentrations of WPN for 24 h.

Snail Population	Mortality of Snails in Different Concentrations of WPN								LC_50_ (mg/L)
	1 mg/L	0.5 mg/L	0.25 mg/L	0.125 mg/L	0.063 mg/L	0.032 mg/L	0.016 mg/L	0.008 mg/L	
Weishan	100	100	90	20	15	5	0	0	0.1436
Shimen	100	100	80	80	50	10	0	0	0.0770
Linli	100	95	75	15	10	5	5	0	0.1708
Ruanjiang	100	95	95	70	25	0	0	0	0.0981
Nanxian	100	80	65	20	5	0	0	0	0.2177
Gong’an	100	100	63.33	3.33	0	0	0	0	0.2222
Jingshan	100	100	93.33	6.67	6.67	0	0	0	0.1684
Wucheng	100	100	100	83.33	26.67	6.67	3.33	0	0.0770
Pengze	100	96.67	76.67	20	0	0	0	0	0.1856
Duchang	100	100	100	46.67	20	0	0	0	0.1115
Yongxiu	100	100	100	80	30	15	0	0	0.0743
Nanchang	100	100	100	85	15	5	0	0	0.0854
Xuancheng	100	96.67	66.67	0	0	0	0	0	0.2285
Dongtai	100	100	90	5	0	0	0	0	0.1830
Zhenjiang	100	100	77	27	20	0	0	0	0.1497
Pinghu	100	100	86.67	23.33	3.33	3.33	0	0	0.1575
Yinxi	100	100	100	10	0	0	0	0	0.1649

The *O. hupensis* snails derived from 16 sampling sites (the number of snails sampled from Shimen county of Hunan province was not enough, due to death, to be used for assessing the mortality following immersion in WPN for 48 h) were employed to test the efficacy of WPN following immersion for 48 h. No snails survived the treatment with WPN at concentrations of 0.5 and 1 mg/L for 48 h, whereas all snails were alive by immersion in WPN at a concentration of 0.008 mg/L for 48 h. Immersion in WPN at concentrations of 0.25, 0.125, 0.063, 0.032 and 0.016 mg/L for 48 h resulted in 95%–100%, 10%–100%, 0%–55%, 0%–10%, and 0%–5% mortalities of snails, respectively (Table 3). 

**Table 3:** Comparison of mortality rates and LC_50 _values of *Oncomelania hupensis* snails sampled from different regions of China following immersion in various concentrations of WPN for 48 h.

Snail Population	Mortality of Snails in Different Concentrations of WPN								LC_50_ (mg/L)
	1 mg/L	0.5 mg/L	0.25 mg/L	0.125 mg/L	0.063 mg/L	0.032 mg/L	0.016 mg/L	0.008 mg/L	
Weishan	100	100	100	70	10	5	0	0	0.1015
Shimen ^**a**^	–	–	–	–	–	–	–	–	–
Linli	100	100	100	25	20	5	5	0	0.1208
Ruanjiang	100	100	100	75	35	0	0	0	0.0825
Nanxian	100	100	95	75	15	0	0	0	0.0981
Gong’an	100	100	100	60	10	0	0	0	0.1088
Jingshan	100	100	100	40	3.33	3.33	0	0	0.1309
Wucheng	100	100	100	90	13.33	3.33	0	0	0.0864
Pengze	100	100	100	63.33	10	0	0	0	0.1063
Duchang	100	100	100	80	33.33	0	0	0	0.0806
Yongxiu	100	100	100	100	55	30	0	0	0.0490
Nanchang	100	100	100	100	30	0	0	0	0.0718
Xuancheng	100	100	96.67	16.67	0	3.33	0	0	0.1575
Dongtai	100	100	100	25	0	0	0	0	0.1487
Zhenjiang	100	100	97	57	13	10	0	0	0.1037
Pinghu	100	100	96.67	23.33	0	0	0	0	0.1539
Yinxi	100	100	100	10	5	0	0	0	0.1593

Note: **^a^** The number of *O. hupensis* snails sampled from Shimen county of Hunan province is not enough to be used for assessing the mortality following immersion in WPN for 48 h.

The 48 h WPN LC_50_ values for the *O. hupensis* snails derived from 16 sampling sites in China ranged from 0.049 to 0.1593 mg/L, and no significant difference was detected by the Kolmogorov-Smirnov test (*p* = 0.197).

## 4. Discussion

In China, the description of schistosomiasis japonica dates back more than two millennia [2]. Since the control efforts initiated in the 1950s, snail control has been a key part of the National Schistosomiasis Control Program of China [8,12], and many approaches have been used for the control of the intermediate host snails, including environmental improvement, physical methods, building trees, molluscicide treatment and biological control [11]. Among all these strategies, molluscicide treatment is the most widely used method for snail control due to the wide application coverage, easy procedure and fast action [20]. A recent meta-analysis revealed that it is necessary to continuously apply molluscicidal treatments more than twice a year in the field to consolidate the schistosomiasis control achievements gained [21], and different molluscicidal treatment strategies should be utilized at different stages of the control programme to maximize cost-effectiveness [20]. 

Since 1950s, many chemicals have been tested for molluscicidal activity and several agents have been used for snail control in the schistosomiasis-endemic fields of China, including sodium pentachlorophenate, bromoacetamide, nicotinanilide, calcium cyanamide, niclosamide, META-Li, and so on [22,23,24,25]. In addition, some plants have been screened and tested for molluscicidal activity against *O. hupensis* in laboratories [26,27,28,29,30]. Since 1992 when the World Bank Loan Project for Schistosomiasis Control was initiated in China, WPN was introduced and has replaced other molluscicides to become the only molluscicide now used for snail control in the endemic areas of China [15]. Following the repeated, long-term, extensive use for more than two decades, there is a concern about the potential development of resistance to niclosamide in *O. hupensis* snails [16]. It is therefore of great importance to monitor the molluscicidal activity of niclosamide against the *O. hupensis* snails in regions where this is currently the only available chemical that is widely used in China. 

WPN at a dose of 1 mg/L is recommended for snail control in the field [15]. Therefore, drug solutions at concentrations of 1, 0.5, 0.25, 0.125, 0.063, 0.032, 0.016 and 0.008 mg/L were designed for the molluscicidal tests in the laboratory. Our findings showed that all *O. hupensis* snails were dead following immersion in 1 mg/L WPN for 24 and 48 h or in 0.5 mg/L WPN for 48 h, and only 0%–20% survived the treatment with WPN at a concentration of 0.5 mg/L for 24 h. In addition, the 24 h WPN LC_50_ values for the *O. hupensis* snails derived from 17 sampling sites in China ranged from 0.0743 to 0.2285 mg/L, with no significant differences were detected (*p* = 0.2), and the 48-h WPN LC_50_ values for the *O. hupensis* snails derived from 16 sampling sites in China ranged from 0.049 to 0.1593 mg/L, with no significant differences detected (*p* = 0.197). The results from this study demonstrate that the molluscicidal activity of niclosamide against *O. hupensis* is still high in the main endemic areas of China after more than two decades of repeated, extensive use. This is important information for both the public health workers and health policy makers in the field of schistosomiasis control, considering that niclosamide, the currently only chemical of choice for snail control, plays an essential role in the current Chinese National Schistosomiasis Control Program of China. 

It has been found that the environmental factors including temperature, vegetation, sunlight, soil and rainfall, the quality and concentration of the chemicals, as well as the technical skills may affect the molluscicidal actions in the endemic foci [11], whereas in the laboratory, the volume of drug solution [31], and the quantity, laboratorial breeding duration and the sampling time of snails used for molluscicidal test [32,33,34] are reported to affect the evaluation of the molluscicidal actions in the laboratory. Currently, a single WPN dose (1 mg/L) is employed for the field snail control in China [15], however, the variations in snail habitats, climate, temperature and rainfall may affect the concentration and duration of snail contact with niclosamide, thereby resulting in various molluscicidal efficacies. It is suggested that the dose of molluscicides should be appropriately adjusted based on the actual field settings, and temperature, climate and rainfall should be taken into account during the snail control in the field, so as to achieve the optimal molluscicidal efficacy.

## 5. Conclusions

The results indicate that there is no regional variation in the current susceptibility to niclosamide in *O. hupensis* populations, and the current activity of niclosamide against *O. hupensis* snails in China appears satisfactory; however, it does not mean that resistance cannot occur nor that in different geographical regions the response of *O. hupensis* snails will be the same. We, therefore, should not reduce our vigilance to the possible development of resistance to niclosamide in the intermediate host snails. Further periodical studies monitoring both the sensitivity of *O. hupensis* to the chemical and the development and epidemiology of niclosamide resistance of different geographical isolates of *O. hupensis* in China are still required, which would be of great significance for the elimination of schistosomiasis japonica in China.
